# Implantable Photothermal Agents based on Gold Nanorods-Encapsulated Microcube

**DOI:** 10.1038/s41598-018-31793-9

**Published:** 2018-09-12

**Authors:** Hyun June Moon, Minhee Ku, Hyunjee Lee, Nara Yoon, Jaemoon Yang, Ki Wan Bong

**Affiliations:** 1 Department of Chemical and Biological Engineering, Korea University, Seoul, 02841 Republic of Korea; 2 Department of Radiology, College of Medicine, Yonsei University, Seoul, 03722 Republic of Korea; 3 Systems Molecular Radiology, Yonsei University, Seoul, 03722 Republic of Korea; 4 Research Institute of Radiological Science, Yonsei University, Seoul, 03722 Republic of Korea

## Abstract

Gold nanorods (GNRs) are of great interest in cancer therapy given their ability to ablate tumor cells using deep tissue-penetrating near-infrared light. GNRs coated with tumor-specific moieties have the potential to target tumor tissue to minimize damage to normal tissue. However, perfect targeting is difficult to achieve given that nanoparticles could be broadly dispersed inside the body. Moreover, interaction between targeting groups and biological molecules could lower targeting abilities, resulting in off-target accumulation which might produce nanotoxicity. Here we introduce GNR-encapsulated microcubes (GNR@MCs) that can be utilized as implantable photothermal agents. GNR@MCs are created by encapsulating GNRs in polymeric networks via stop flow lithography (SFL), a one-phase synthesis technique which allows for creation of surfactant-free, uniform particles, and injection of GNR@MCs into the body after a simple rinse step. GNRs are highly packed and firmly encapsulated inside MCs, and entrapped GNRs exhibit optical properties comparable to that of unbound GNRs and photothermal efficiency (58%) in line with that of nano-sized agents (51–95%). Photothermal ablation in murine models is achieved using GNR@MCs stably implanted into the tumor tissue, which suggests that GNR@MCs can be a safe and effective platform for cancer therapy.

## Introduction

Noble metal nanoparticles are of great interest in cancer therapy since their distinctive localized surface plasmon resonance (LSPR) can be used to kill tumor cells via photothermal ablation. Heat is produced by strong oscillation of free electrons at the nanoparticle surface under irradiation of a certain wavelength of light, and the elevated local temperature causes irreversible damage to neighboring tumor cells. Photothermal ablation of tumor cells located in deep tissues is challenging since light energy normally decreases when passing through complex biological systems, leading to insufficient heat production at the tumor site. Near-infrared (~700–1,200 nm, *e*.*g*., NIR) wavelengths largely comprise the “optical window” for photothermal therapy since NIR light shows minimized absorption in biological systems^1^.

To make nanoparticles produce heat triggered by NIR light emission, the LSPR of particles has been adjusted by changing the morphology (*i*.*e*., size and shape) and compositions of nanoparticles and the chemical environment (*i*.*e*., pH) of their surroundings^2–5^. For spherical gold nanoparticles (*e*.*g*., gold nanospheres), LSPR can be tuned by adjusting particle size, but the limited tuning range makes it nearly impossible for particles to exhibit LSPR under NIR light^6^. Changes in morphology (*e*.*g*., shell, cage, and rod) can provide drastic tuning of LSPR, either by coupling of surface plasmons or generating two different modes of resonance^7–10^. Gold nanoshells, nanocages, and nanorods have been considered promising tools for photothermal agents, with gold nanorods (GNRs) receiving significant attention due to: (1) the LPSR of GNRs can be tuned by simply adjusting the aspect ratios (longitudinal length/transverse length), and (2) the broader cross-sectional area of GNRs renders higher light energy absorbance, resulting in enhanced photothermal heating effects^8^.

Surface engineering of nanoparticles is another critical design parameter as it relates to function in the human body without producing toxic effects. Among the noble metal nanoparticles that exhibit LSPR, gold nanoparticles are of great interest due to non-toxicity, non-immunogenicity, and high tissue permeability without interfering with cell functionality^11–13^. GNRs can be coated with poly(ethylene glycol) (PEG) and functionalized with peptides, aptamers, and antibodies to make them biocompatible and possible for targeted delivery to various tissue sites^14–16^. However, even the highly selective GNRs are not free from nanotoxicity issues since their small size makes them susceptible to broad dispersion in the body and unintended cellular uptake. Targeting efficiency can also decrease significantly due to unexpected interactions between targetable agents and biomolecules inside complex biological systems^17–22^ and accumulation kinetics and long-term toxicity of GNRs in the body that have not been fully studied^23,24^. Therefore long-term toxicity issues cannot be neglected, and solutions for such potential problems are highly required before using GNRs or other nanoparticles for tumor therapy.

On the other hand, GNR(or other nanoparticles)-embedded hydrogel microparticles show proper aspects—their water-like environment allows near-infrared light to penetrate through the particle volume, the polymer networks can firmly encapsulate nanomaterials, and the networks can be made up of biocompatible polymer (such as poly(ethylene glycol); PEG). In addition, the particles generated via flow lithography have been of a great interest in a variety of biological fields such as drug delivery^25,26^, tissue engineering^27,28^, and bio-molecule sensing^29^, based on the facts that this technique enables synthesis of microparticles with complex morphologies and well-tailored compositions, with all the beneficial aspects of the hydrogel microparticles stated above. Therefore, GNR-embedding microparticles synthesized via flow lithography could help clear the potential nanotoxicity, while not significantly compromising the photothermal therapeutic effects of GNRs.

Here we introduce GNR-encapsulated microcubes (GNR@MCs) in which GNRs are highly packed into the polymeric networks via stop flow lithography (SFL). A precursor solution in a polydimethylsiloxane (PDMS) channel is exposed to UV light through a micro-patterned photomask, and uniform particles are generated with dimensions controlled by channel topography^30,31^. Particles can be readily injected into the body after a simple rinse step since SFL is a one-phase synthesis method that does not require use of organic solvents or chemical surfactants which can induce cytotoxic effects^32^. GNRs can be encapsulated in the cross-linked polymer networks since GNRs exhibit two absorbance peaks (~514 and ~753 nm) far away from UV light (~365 nm) used in SFL. Since photo-crosslinking reactions are not compromised, most of the GNRs (~99.4%) initially suspended in the precursor solution can be encapsulated in microcubic particles. GNR@MCs can generate significant photothermal heating effects since NIR light can easily pass through the water-like environment of the microcubes. A sufficient number of GNRs trapped inside the porous networks absorb NIR light and generate heat that can be transferred through the microcubic particle, confirmed by the relatively high photothermal transduction efficiency (~58%) of GNR@MCs. Microcubic particles with uniform size (~50 μm) can be implanted into specific tissue sites and generate photothermal heating effects comparable to that of unbound nanorods.

## Results and Discussion

### Design and Synthesis of GNR@MCs

**Figure 1:**
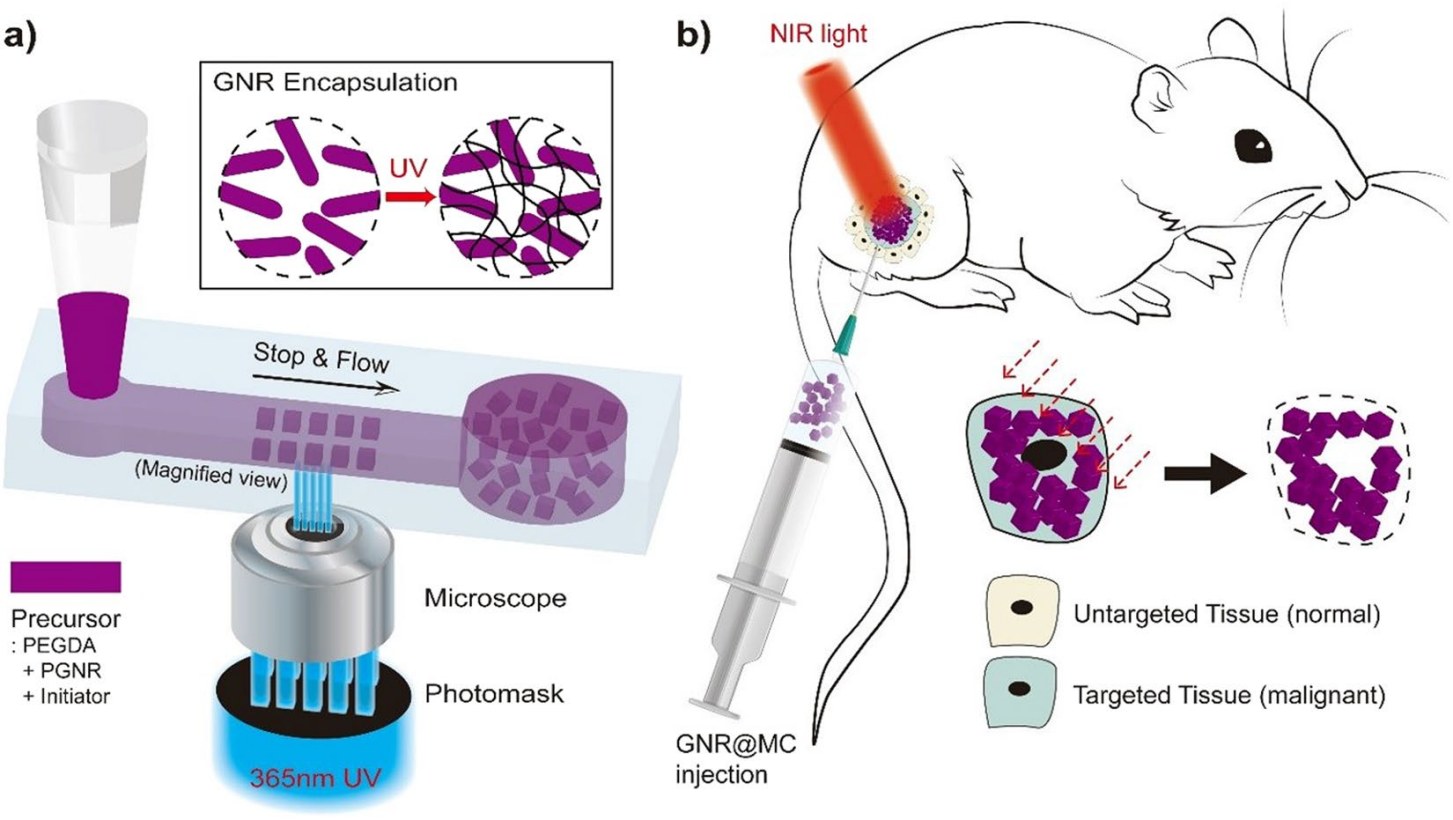
A Schematic illustration of implanted photothermal therapy. (**a**) synthesis of gold nanorod-encapsulated microcube (GNR@MC) via stop flow lithography (SFL) and (**b**) application of GNR@MC as implanted photothermal agents.

**Figure 2:**
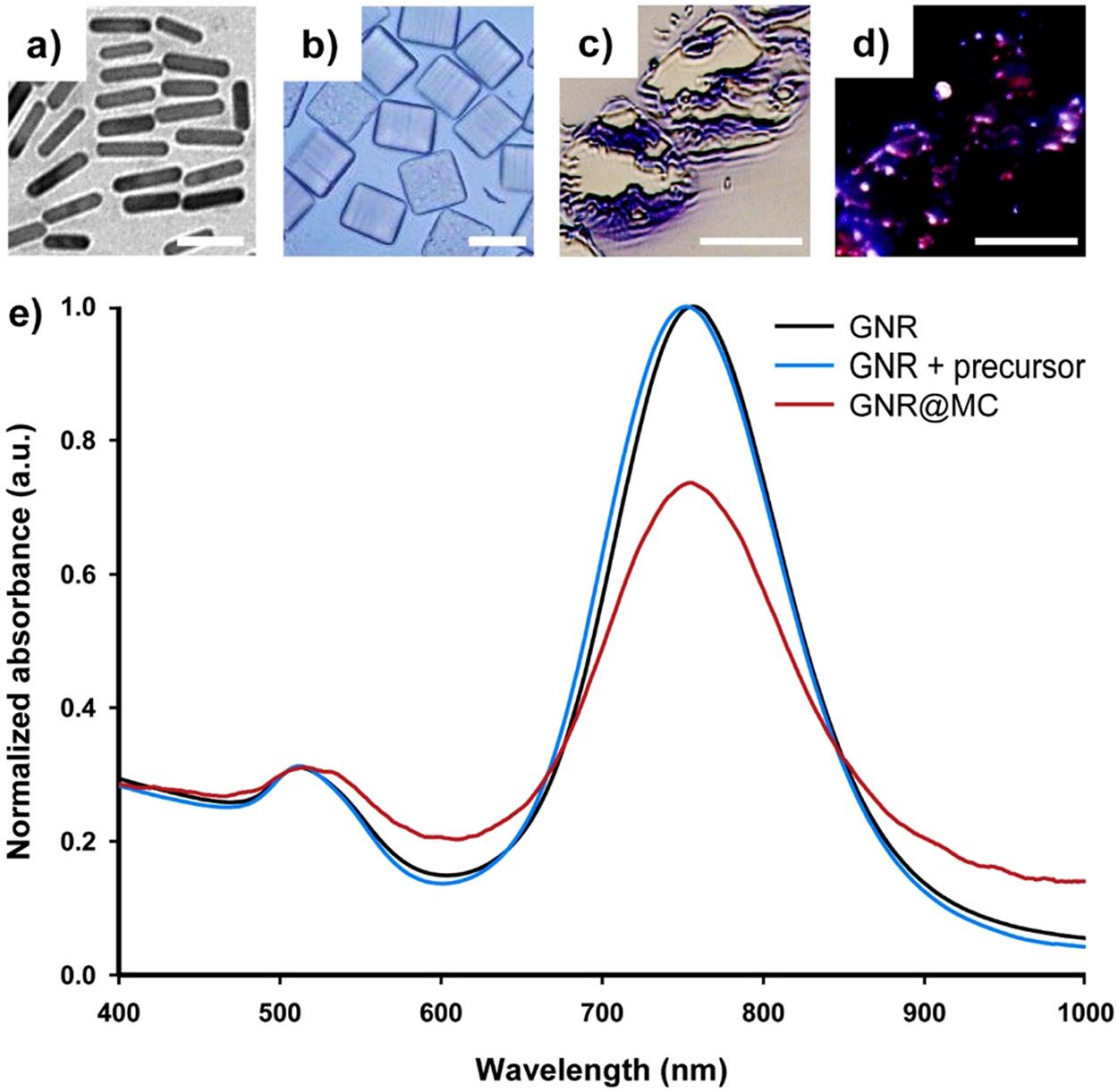
Optical characteristics of GNR and GNR@MC, and images. (**a**) transmission electron microscopic (TEM) image of GNRs, (**b**) bright field image of GNR@MCs, (**c**) bright field image of sliced GNR@MCs, (**d**) dark field image of sliced GNR@MCs, and (**e**) Normalized absorbance spectra for GNR dispersed water (black), GNR dispersed precursor solution (blue), and GNR embedded in the polymeric network of GNR@MCs (red). Scale bars are 20 nm, 50 µm, 50 µm, and 50 µm, respectively (from **a** to **d**).

Poly (ethylene glycol) (PEG) was selected as the polymer network for the fabrication of GNR@MCs due to these properties: (1) minimal toxic effects in biological systems, (2) optically transparent for NIR light, and (3) heat produced by LSPR can be transferred through water-like medium. Cubic microparticles with a side length of ~50 μm were synthesized to minimize particle movement away from the injection site. Following SFL, highly monodisperse GNR@MCs were recovered after a rinse step to remove unreacted oligomer and photo-initiator molecules (Figs 1a and 2b). A homogenous distribution of GNRs in an individual MC was achieved, which maximizes surface area to produce enhanced photothermal effects. PEGylated GNRs (PGNRs) were used given the similarity with the PEG-based precursor solution and surface covering materials of GNRs. PGNRs in the PEG-based precursor solution showed a homogeneously mixed state, without significant flocculation over 6 hours (Supplementary Fig. S1a). In contrast, GNRs showing cetyltrimethylammonium bromide as the surface-capping materials showed rapid flocculation, characterized by a formation of remarkable aggregates (Supplementary Fig. S1b).

### Characterization of GNR@MCs

#### Morphology of GNRs and GNR@MCs

The morphology of GNRs and GNR@MCs was observed through transmission electron microscope (TEM) images and optical microscope images. GNRs had a longitudinal length of ~43.18 nm (with a coefficient of variance; CV = 0.08) and a transverse length of ~10.3 nm (CV = 0.084), resulting in an aspect ratio of ~4.2 (Fig. 2a). Using bright field microscopy, GNR@MCs had a side length of ~50 μm (CV = 0.014) and a height of ~42.5 μm (CV = 0.016) (Fig. 2b). To verify GNR encapsulation in polymer network, qualitative analysis using dark field microscopy was conducted. However, an individual GNR@MC was too large for the microscope to focus the incident light from the light source. Thin films of GNR@MCs using a micro-sawing process were then prepared to determine an appropriate sample height before conducting dark field microscopy. Using bright field images as shown in Fig. 2c and dark field images in Fig. 2d, loading of GNRs in MCs was shown qualitatively based on GNRs encapsulated in MCs displaying a reddish color. The reddish color did not appear to be broadly dispersed throughout the samples, but the harsh nature of the micro-sawing process likely distorted the exposed regions of the GNR@MCs. Since the homogeneous precursor solution was cross-linked immediately (~0.1 s), uniform distribution of GNRs inside the MCs was highly likely.

#### GNR encapsulation efficiency inside the GNR@MCs

The GNR encapsulation efficiency of MCs was quantitatively analyzed since it plays a key role in maximizing therapeutic efficacy while minimizing nanoparticles’ leakages out of the MCs. For GNR@MC synthesis, the most proper precursor compositions were selected. Given that GNRs’ transverse (smaller) length shows a Gaussian distribution (mean~10.28 nm, standard deviation~0.87 nm), the average pore diameter has to be as small as 7.67 nm for a nearly perfect encapsulation (the theoretical encapsulation efficiency is greater than 99.8% when $\frac{{\rm{pore}}\,{\rm{size}}-10.28}{0.87} < -\,3$). Therefore, particles were synthesized using precursor solution consisted 60% (v/v) cross-linkable species (*i*.*e*., PEG(700)diacrylate), which perfectly capture materials with the radius of gyration larger than ~4 nm^33,34^. To experimentally confirm whether MCs encapsulate GNRs perfectly, the theoretical embedded mass was estimated. The precursor solution contained ~2.4302 × 10^−2^ mg/ml suspended GNRs (details of the calculation are shown in the Supplementary Information). The ideal encapsulation mass per MC can yield ~2.4302 × 10^−2^ mg/ml of GNRs embedded inside an unoccupied volume of 1.063 × 10^−7^ ml (based on an individual GNR@MC with 50 μm side length and 42.5 μm height), resulting in ~2.583 pg of GNRs encapsulated per MC. The actual amount of GNRs in an individual MC was measured by using inductively coupled plasma atomic emission spectroscopic (ICP-AES) analysis. In 1 mL of GNR@MC solution with an Au concentration of 142.3 mg/L, there were 52,268 GNR@MCs (with a volume of ~1.063 × 10^−7^ ml) meaning that approximately 2.561 pg of Au was present in an individual MC. Comparing these two values (ideal and measured), the GNR-loading efficiency was estimated as ~99.14%, with a tolerable error that can be accompanied by measurement errors. Further, to test whether GNRs could leak out of MCs, GNR@MCs were submerged in water for specific time periods or temperature settings, and the supernatant was extracted and analyzed for two absorbance peaks (~514 and 753 nm) representative of GNRs. The difference in absorbance at both GNR-specific peaks across the samples were negligible, which indicates that GNRs are firmly encapsulated in the polymer networks (Supplementary Fig. S2).

#### GNR encapsulation yield during the GNR@MC synthesis

GNR encapsulation yield, which is the effective GNR loading inside the MCs throughout synthesis process, was maximized by optimizing the mask design and operating conditions of flow lithography. First, to enable continuous synthesis of uniform MCs, the distance between square patterns of the photomask was selected as 25 μm to prevent particles from being linked to each other due to potential cross-linking reaction. Second, the precursor flow rate was optimized to migrate the polymerized MCs sufficiently to prevent the overlapping between the MCs, but not too large to avoid the overuse of the precursor solution (Supplementary Fig. S3). Here, the GNR encapsulation yield during flow lithography can be estimated by comparing the precursor used for synthesizing GNR@MCs with the one not used and rinsed after MC synthesis. In the synthesis conditions mentioned above (i.e., pattern distance and precursor flow rate), 15 GNR@MCs were synthesized per a cycle of 0.8 seconds (flow 300 ms, stop 400 ms, lithography 100 ms), while consuming the precursor volume of ~100 μl in ~80,000 seconds. Therefore, 150,000 GNR@MCs having the volume of ~1.0625 × 10^−4^ μl (50 × 50 × 42.5 μm^3^) were generated using 100 μl precursor solution, indicating the yield of ~0.1594 (1.0625 × 10^−4^ μl/MC × 150,000 MC/100 μl). Another estimation has been taken based on analyzing GNR@MCs’ movement along the precursor flow. Supplementary Fig. S3b shows the distance GNR@MC migrate, and it was assumed that particles do not stick to the channel wall and move along the precursor streamlines. The MC at the centerline moves around 600 μm per 300 ms (flow time), suggesting the maximum velocity of ~2 μm/ms and flow rate of ~11,250 × 10^−6^ μl/s (~0.8 × 11,250 × 10^−6^ μl/cycle(0.8 s)). On the other hand, the precursor used for GNR@MC synthesis (effective volume per cycle) is ~15 × 1.0625 × 10^−4^ μl/cycle, indicating the precursor yield of ~0.1771, which agrees well with the estimated yield mentioned above. The un-embedded GNRs suspended in the un-polymerized precursor solution can be recovered simply by rinsing and centrifugation (Supplementary Fig. S3c). Moreover, the recovered GNRs show similar optical properties compared with that of intact GNRs (Supplementary Fig. S3d), suggesting that the yield mentioned above can be greatly increased via recycling the GNRs.

#### Optical properties of the GNR@MCs

To assess whether encapsulated GNRs exhibit consistent optical properties compared with that of GNRs dispersed in the liquid (in water or the precursor solution), UV-vis-NIR spectroscopy was used to measure the relative absorbance spectra of GNR@MCs, GNRs in water, GNRs in the precursor solution, and GNR@MCs. The two absorbance peaks (~514 nm and ~753 nm) were conserved for GNR@MCs and GNRs in water, while the ~753 nm peak exhibited some decay for encapsulated GNRs Fig. 2e. The conservation of two peaks indicates the similarity in optical characteristics between GNRs in suspension (water or precursor solution) and GNR@MCs. Encapsulated GNRs exhibited less absorbance, possibly due to being unable to freely reorient like GNRs in suspension^35^. The decrease in NIR peak could indicate the reduced potency of encapsulated GNRs due to reduced NIR energy absorption, which in turn reduce photothermal heating effects. Despite a lower potential of GNR re-orientation in the polymer network, GNR@MCs can exhibit comparable photothermal heating effects.

#### Bio-availability of the GNR@MCs

The cell viability of the GNR@MCs was evaluated by quantifying live MDA-MB-231 cells. The viability remained above 95% when they were incubated in the presence of GNR@MC with different cell-to-particle ratios (Supplementary Fig. S4), which indicates that GNR@MCs can be applied to biological conditions.

### Validation of Photothermal Effects of GNR@MCs

#### *In vitro* Photothermal Analysis

**Figure 3:**
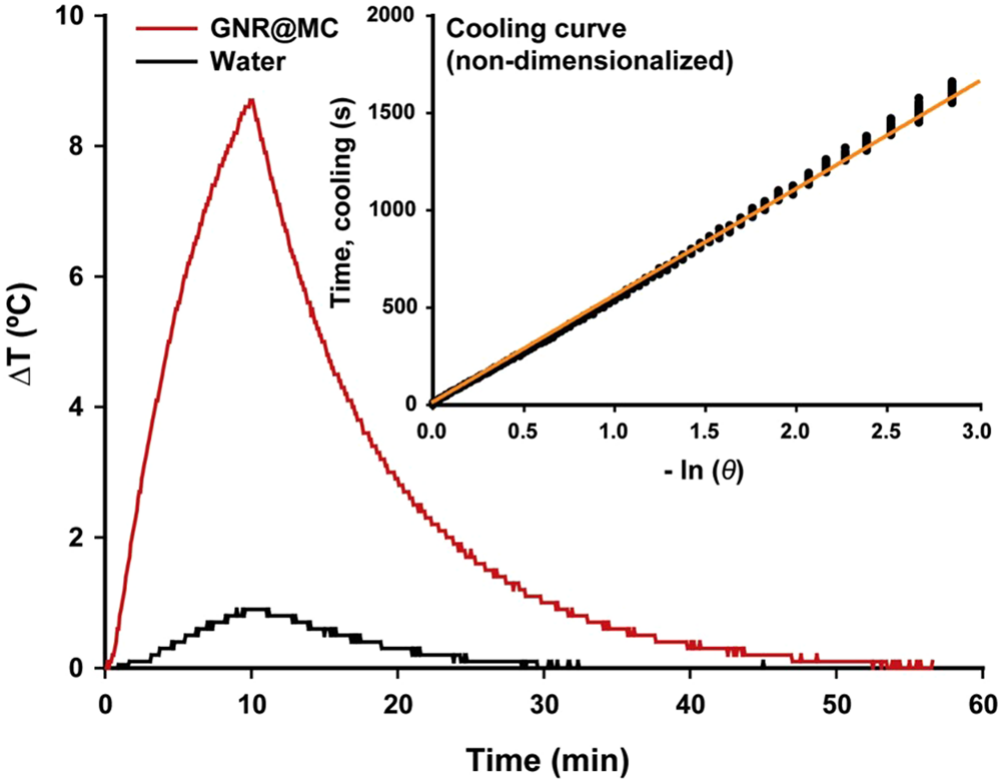
Real-time photothermal heating of GNR@MC. The change in temperature of GNR@MC dispersed solution (red) and water (black) as a function of time. The inset represents the linear relationship between time and −ln(θ) obtained from the laser off the state of GNR@MC. The power intensity of the laser was 20 W/cm^2^, and the irradiation time was 10 minutes. The interpretation and calculation of −ln(θ) are described in the text.

To estimate photothermal transduction efficiency of GNR@MCs, the heating and cooling profile of a GNR@MC suspension in water was measured in real time using a thermocouple (Fig. 3). For a 10-minute heating interval, the temperature elevation of ~0.9 °C was observed for water alone when irradiated with a laser of 20 W/cm^2^ and 2 mm of beam diameter. A temperature elevation of ~8.7 °C was observed for a GNR@MC suspension in water using the same laser settings. The photothermal transduction efficiency of GNR@MC was quantified by analyzing the heat transfer based on the temperature gradient between NIR-exposed area and the unexposed area, according to a modified version of a previous method^36,37^. A heat transfer time constant of τ_s_ = 546 s was determined from the slope of time versus −ln(θ) during the cooling period of GNR@MCs (inset in Fig. 3). The data were truncated at 2550 s to avoid divergence as the logarithmic operand approaches zero. It is known that the evaluation of the time constant with the cooling data can be useful to avoid the effects of thermal gradients during heating^36^. Using the time constant value, *t*_*s*_, absorbance at 808 nm (A^808^ ~0.1974), heat capacity of the system (~8.3628 J/K), and light energy input rate (~0.628 W) the photothermal transduction efficiency of GNR@MC was estimated as 58.0%, which is lower than that of the typical unbound GNRs (~90%)^36,38^. The decrease in the heating efficiency may be due to GNRs being so firmly captured by the MCs that GNRs cannot re-orient to absorb more NIR light energy. However, the efficiency (~58%) can be considered sufficient for photothermal therapy given that other nano-sized photothermal agents typically show efficiencies ranging from 51% to 95%^39^. These results suggest that GNR@MCs can be a potentially effective platform for photothermal therapy.

#### Bio-availability of the GNR@MCs

The cell viability of the GNR@MCs was evaluated by quantifying live MDA-MB-231 cells. MDA-MB-231 cells were grown over 24 h to attach and the culture media was exchanged for serially diluted GNR@MC-contained media with various concentrations. The GNR@MC-treated cells were monitored for 20 h in real time and showed no discernible difference in the morphology of adherent cells compared to GNR@MCs-untreated cells (Supplementary Fig. S4a). Subsequently, the numbers of live cells in three randomly chosen position were counted. Cell count data represented that the cell viability was remained above 95% in each of cell-to-GNR@MC ratios (Supplementary Fig. S4b). This results indicates that GNR@MCs can be applied to biological conditions.

#### *In vitro* Cancer Cell Ablation

**Figure 4:**
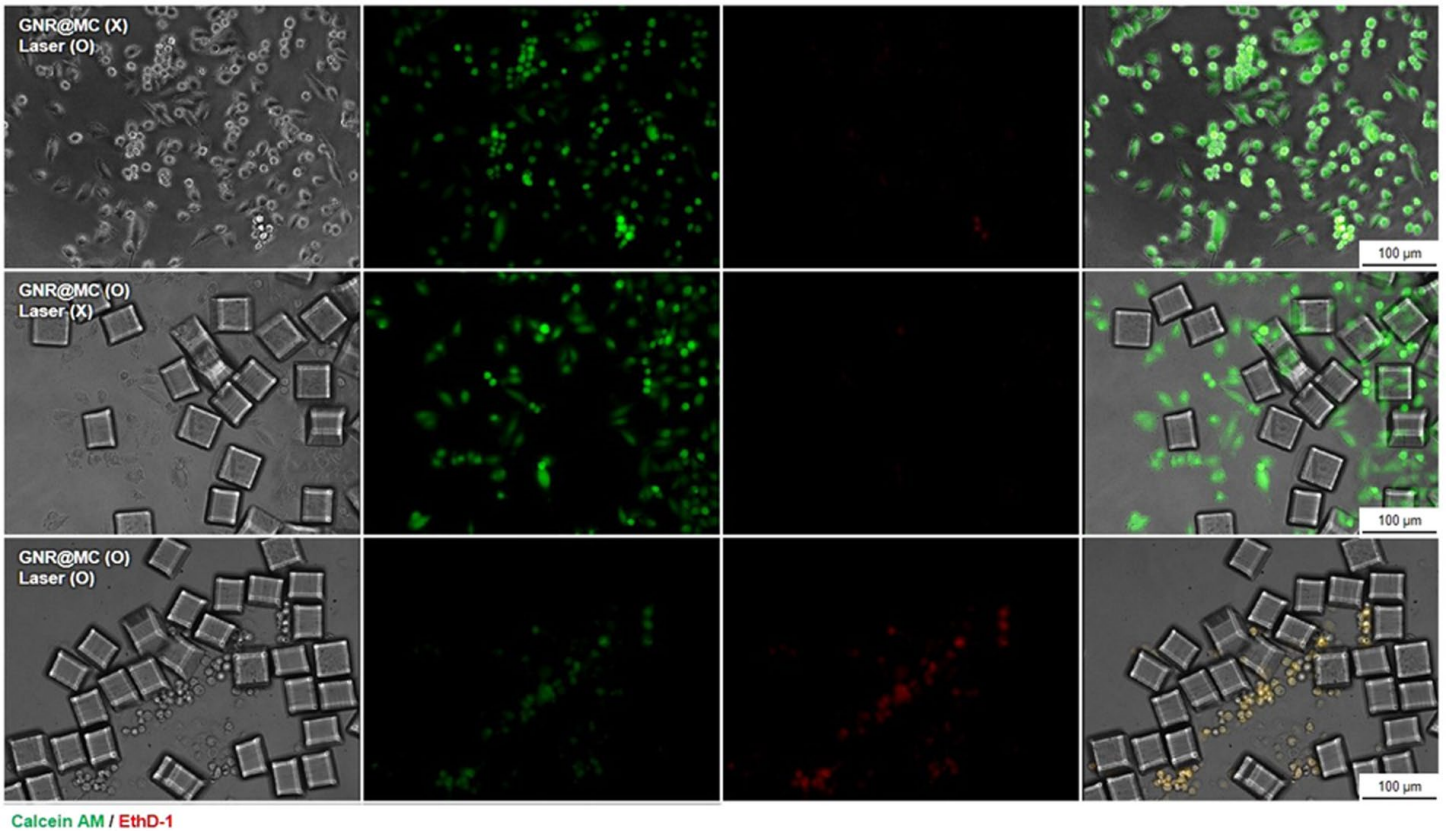
*In vitro* cancer cell ablation test. Live/Dead assay of MDA-MB-231 cells treated with PBS and GNR@MC with or without NIR laser irradiation. MDA-MB-231 cells were co-stained with calcein AM (green, live cells) and ethdium homodimer-1 (EthD-1, red, dead cells) after the NIR laser irradiation.

To verify the GNR@MC-mediated, controlled photothermal ablation of cancer cells, *in situ* cell ablation study was conducted. The cancer cells (MDA-MB-231) were subjected to either GNR@MC co-culture or NIR laser exposure. First, for the cells co-cultured with GNR@MCs, when irradiated under NIR light for 10 minutes, the morphological damage of the cells was observed around GNR@MC on phase images (Supplementary Fig. S5). In order to assess the photothermal therapeutic efficiency of GNR@MC on cancer cells, the cells were stained with LIVE/DEAD viability/cytotoxicity staining kit which can distinguish live cells (green fluorescence) from dead or dying cells (red or yellowish fluorescence). In Fig. 4, MDA-MB-231 cells in the presence of GNR@MCs and under NIR light irradiation clearly exhibit red fluorescence as a result of photothermal damage resulting in the cell death. However, MDA-MB-231 cells exposed to NIR irradiation without introducing GNR@MCs or co-cultured with GNR@MC without irradiating NIR light expressed green fluorescence signals only. Therefore, the cancer cells, when subjected to both the GNR@MC co-culture as well as NIR light exposure, get damages due to the photothermal effects.

#### *In vivo* Photothermal Ablation

**Figure 5:**
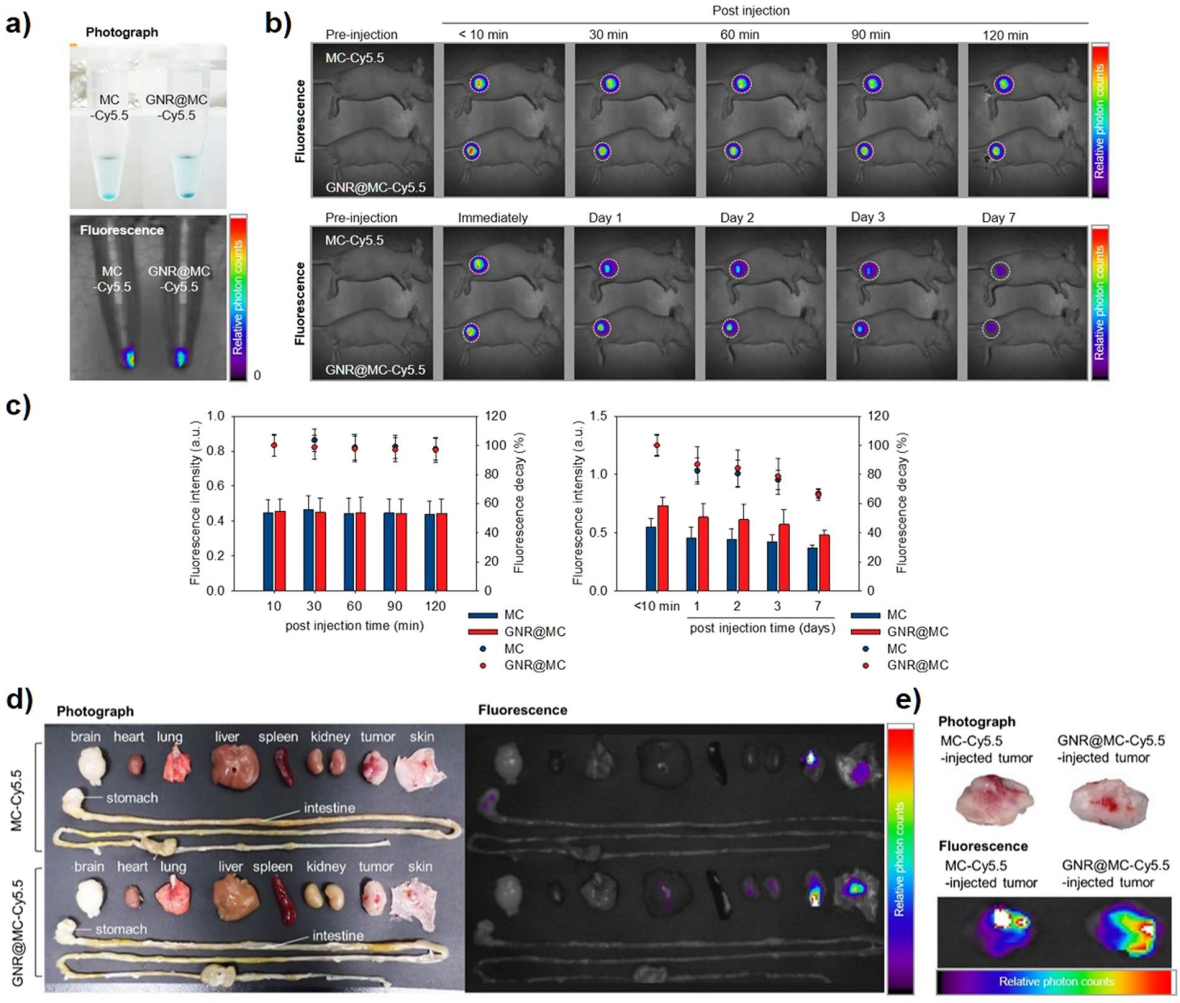
*In vivo* stability test of Cy5.5-conjugated MC and GNR@MC. (**a**) Photographs and fluorescence images of MC-Cy5.5 and GNR@MC-Cy5.5. (**b**) *In vivo* optical fluorescence images of MDA-MB-231 tumor-bearing mouse after the implantation of MC-Cy5.5 or GNR@MC-Cy5.5 at various time intervals, respectively. A white dashed boundary indicated the tumor site. (**c**) Graph of relative photon counts obtained from (**b**) referred as fluorescence intensity (bar graph, left axis). Fluorescence decay represented as a dot plot (Right axis). Error bars mean standard deviation. (**e**) *Ex vivo* NIR fluorescence images of extracted organs (brain, heart, lung, liver, spleen, kidney, tumor and skin covering tumor) and tumor at day 7 post-injection of MC-Cy5.5 and GNR@MC-Cy5.5. (**f**) Photograph and fluorescence images of extracted tumor tissues.

**Figure 6:**
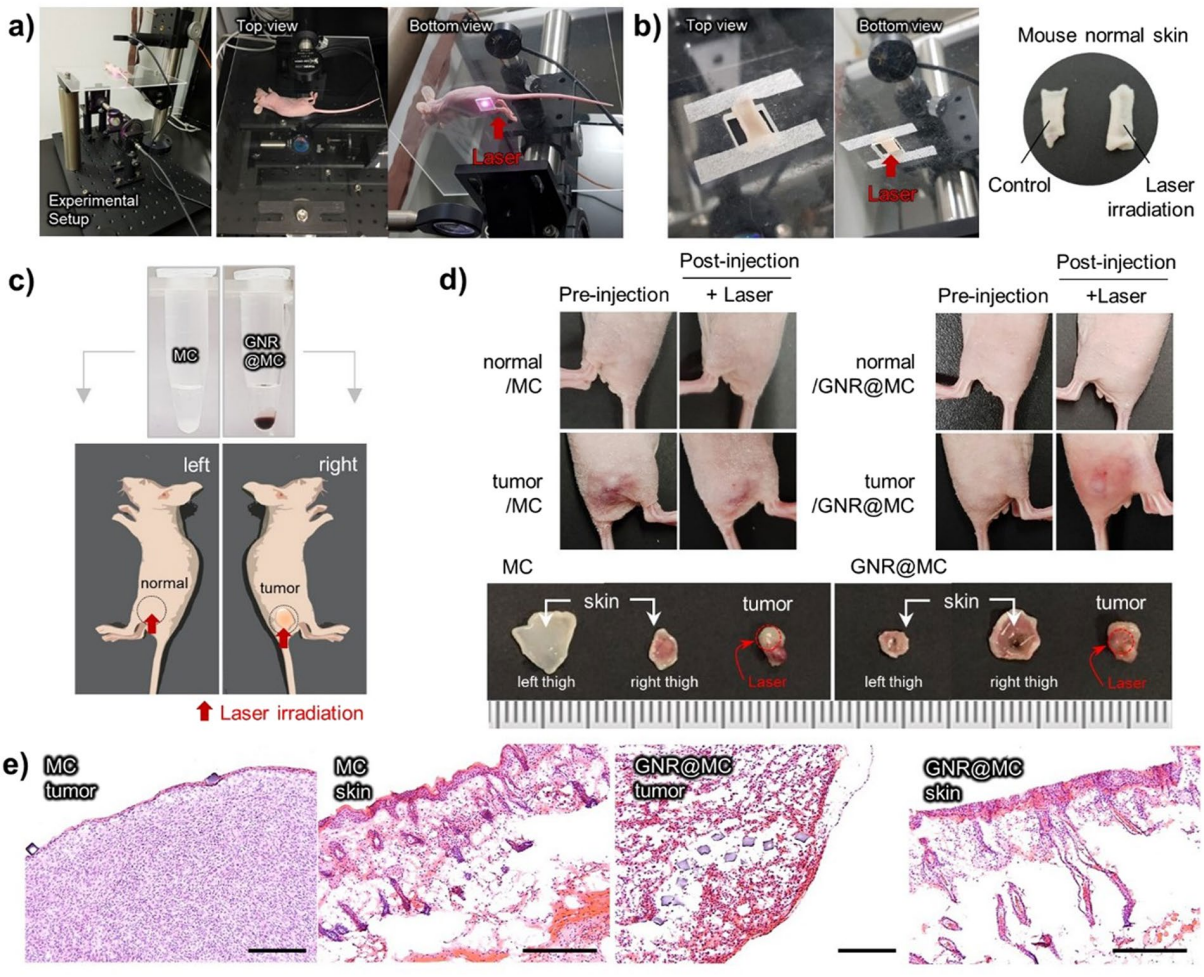
*In vivo* photothermal ablation effect of GNR@MC. (**a**) Photographs of irradiation setting for the photothermal ablation. (**b**) Photographs of extracted mouse normal skin from Balb/c nude mouse; no exposure to laser (left, control) and after exposure to laser (right, laser irradiation). The laser power and irradiation time did not affect to the mouse skin (20 W/cm^2^, 30 min). (**c**) Photographs of MC and GNR@MC solutions (upper) and schematic images of MDA-MB-231 tumor bearing mouse (lower). The MCs were injected to normal skin (left thigh) and GNR@MCs were injected to tumor-forming site (right thigh). (**d**) Photographs of mouse thigh after laser irradiation and extracted skin and tumor after 30 min laser irradiation. (**f**) H&E staining images for tumor-bearing mouse model treated with MC or GNR@MC. Scale bar means 0.5 mm.

To investigate the potential use of GNR@MCs as implanted photothermal (iPT) agents, we tested whether GNR@MCs are implanted without significant movement out of the injection site and show photothermal tumor ablation effects. First, to confirm the location of the implanted GNR@MCs, Cy5.5-conjugated GNR@MCs (GNR@MC-Cy5.5) were prepared. The Cy5.5 dye shows luminescence under NIR light, which enables deep tissue imaging to visualize the location of GNR@MCs (absorbance and luminescence spectra are shown in Supplementary Fig. S6). Additionally, to avoid fluorescence quenching due to NIR absorption by highly crowded GNRs inside the GNR@MCs, Cy5.5-conjugated MCs without GNRs were also prepared. Using GNR@MC-Cy5.5 and MC-Cy5.5, we conducted optical fluorescence imaging in tumor-bearing mice to understand bio-distribution of the MCs. The bio-distribution of the MC-Cy5.5 and GNR@MC-Cy5.5 were observed for 7 days after post-injection of MCs into the proximal thigh for MDA-MB-231 orthotopic xenograft model. After the implantation of MC-Cy5.5 and GNR@MC-Cy5.5, the fluorescence intensity of Cy5.5 dye was fairly strong in a short time frame (up to 120 min post-injection; Fig. 5b, upper and Fig. 5c, left), whereas the fluorescence showed gradual decrease in a relatively long time frame (day scale up to 7 days; Fig. 5b, lower and Fig. 5c, right). The mice were sacrificed at day 7 following exposure and the organs (brain, heart, lung, liver, spleen, kidney, tumor, skin, stomach, and intestine) of each mouse were extracted for relative comparison of fluorescence signals. As shown in Fig. 5d, the other organs showed no distinct fluorescence signal due to the presence of the MC-Cy5.5 or GNR@MC-Cy5.5 except of the tumor tissues and skin covering tumors (Fig. 5e). The results show that the MCs keep their location, implying that they may not accumulate in undesired sites in the body and persist for a long time at the injection site, which would be beneficial for tumor therapy without potential nanotoxicity. Second, to validate photothermal tumor killing effects of the GNR@MCs, *in vivo* photothermal tissue ablation test was conducted. Figure 6a shows the laser irradiation system used for *in vivo* experiments. The system consisted of a fiber-coupled 808-nm diode laser with a collimating lens, a neutral density filter for attenuation of laser power, and a tilting lens for converting the laser beam path to the vertical direction. The laser can irradiate through the bottom of the sample surface, and the irradiated laser beam is collimated by the focusing lens. We evaluated whether the NIR light damage the skin without the presence of GNR@MCs. Figure 6b shows that NIR light itself could not cause any significant damage at the skin, and further experiments were conducted to confirm the tissue damage caused by NIR irradiation (808 nm for 30 minutes) at different laser intensities (10, 20, and 30 W/cm^2^, Supplementary Figs S7b,c and S8). To evaluate the feasibility of GNR@MCs as photothermal agents, photothermal ablation of skin and tumor cells under the presence of GNR@MC and NIR laser irradiation was observed. MCs without GNRs were injected at the normal region (left thigh) and GNR@MC at the tumor-forming region (right thigh) on MDA-MB-231 tumor-bearing mouse (Fig. 6c). After NIR laser irradiated, the thermal damage of skin tissue and tumor at GNR@MC-injected site was observed compared to MC-injected site (Fig. 6d). To prove an extensive confirmation of the targeted photothermal effect, the histologic sections of skin and tumor tissues were analyzed (Fig. 6e). The H&E slices in the MC-injected group were largely representative of normal skin layer with normal morphology. The MC-injected group showed varying degrees of irregular cell size and shape, indicative of tumor tissue after laser irradiation. In contrast, the GNR@MC-injected group showed a considerable damage at skin and tumor tissues due to the thermal effect GNR@MC under NIR irradiation. In addition, Supplementary Fig. S8 shows that GNR@MCs generate sufficient heating effects which could kill the normal tissues at the thigh and the back. Therefore, GNR@MC can be applied as implantable photothermal agents for precision tumor therapy.

## Conclusion

Here we demonstrate that GNRs can be firmly embedded in the polymeric networks of cube-shaped microparticles and exhibit optical properties comparable to that of GNRs in suspension. Despite GNRs being not able to re-orient to receive maximal NIR light, GNRs were highly packed in the MCs, and therefore LSPR heating effects were not significantly compromised. A murine model was used for GNR@MC implantation into tissue, and tumor tissue ablation was achieved using incident NIR laser. By using Cy5.5-conjugated GNR@MCs and MCs we could validate that the MCs kept their location at the injection site, and the GNR@MCs showed a significant photothermal effects supported by both *in vitro* cell ablation and *in vivo* tissue ablation results. By using the implantable photothermal agents, we can treat tumor tissues in more precise way, potentially circumventing broad distribution of nanoparticles in the body, off-target accumulation in the untargeted tissues, and nanotoxicity issues. Furthermore, we envision that this synthesis approach can be extended to create novel combinations of encapsulated nanomaterials and polymer networks for use in disease diagnostics and therapeutics.

## Methods

### PGNR Synthesis

A modified seed-mediated growth method was used to prepare the monodisperse GNRs. Briefly, to prepare the gold seed solution, 250 μL of 10 mM HAuCl_4_·3H_2_O (Sigma-Aldrich) was added to 7.5 mL of 93 mM hexadecyltrimethylammonium (CTAB; Sigma-Aldrich), and then 600 μL of 10 mM ice-cooled sodium borohydride was added to the mixture with vigorous stirring. The mixture was allowed to react for 2 minutes and stored at room temperature for 4 hours, and then a growth solution was prepared as follows. The 9.5 mL CTAB solution was prepared under vigorous stirring, and then 80 μL of 10 mM silver nitrate (Sigma-Aldrich), 50 μL of 10 mM HAuCl_4_·3H_2_O, 55 μL of 100 mM ascorbic acid (Sigma-Aldrich), and 12 μL of gold seed was added to the prepared CTAB solution and stirred for 30 seconds. The resultant solution was stored at room temperature for 24 hours and centrifuged three times at 15,000 rpm for 30 minutes to remove the excess CTAB molecules and re-dispersed in 5 mL of deionized (DI) water. PEG-coated gold nanorods (PGNRs) were synthesized by a coating of GNRs with carboxymethyl-PEG-thiol (Sigma-Aldrich) as a stabilizer. 50 mg of carboxymethyl-PEG-thiol was added to 5 mL of GNR solution (4.73 mM) and stirred for 48 hours at room temperature. The mixture was centrifuged six times at 15,000 rpm for 30 minutes to remove unbound carboxymethyl-PEG-thiol molecules and re-suspended in 5 mL of DI water.

### PDMS Microfluidic Device Fabrication

Microfluidic devices for SFL were fabricated using soft-lithography. The polydimethylsiloxane elastomer (PDMS; Sylgard 184, Dow Corning) was mixed with a 10% (w/w) curing agent. The PDMS and curing agent mixture were rigorously mixed and poured over the (+) patterned silicon master wafer (SU-8 photoresist, Microchem). After 30 minutes for degassing, the (−) patterned PDMS blocks were molded by baking in an oven at 70 °C for about 12 hours. The fully-cured PDMS (−) pattern was detached from the master wafer. The inlet for the precursor injection and outlet for particle recovery were perforated using 1.0 mm and 10.0 mm punches (Miltex), respectively. The PDMS mixed with 10% (w/w) curing agent was coated to glass slides for the bottoms of the microfluidic devices. The thin-coated glass slides were partly cured at 70 °C for 25 minutes. The PDMS (−) patterns with open inlet and outlet were attached to the pre-mentioned bottoms and cured in the oven for at least 6 hours.

### Photopolymerization Setup

Devices were mounted on an inverted microscope (Axiovert 200, Zeiss) equipped with a UV (365 nm) light emitting diode (LED; Thorlabs) as a light source for the photopolymerization. A square patterned photomask (50,000 dpi, Hanall Technology) was mounted into the optical sockets of the microscope. The precursor flow was controlled by a pressure regulator (ITV0031-3BL, SMC). Flow-Stop-Lithography procedure was controlled in synchronized manners to synthesize microcubes automatically. The flow-lithography time (flow; 200 ms, stop; 400 ms, lithography; 100 ms) and pressure (30 kPa) were controlled by a LabVIEW (National Instruments, TX, USA) script.

### GNR@MC Synthesis

Figure 1a shows the procedure of particle synthesis. During SFL, GNR@MCs were synthesized by shining a burst (100 ms) of 365 nm UV light after precursor flow stopped in a precisely synchronized manner (explained in the previous part). The PGNR solution was 3.5 fold concentrated by centrifugation (Eppendorf) in 13,200 rpm for 55 min to synthesize MCs with concentrated GNRs. Each GNR@MC comprised 60% (v/v) PEG700DA (Sigma-Aldrich) as multifunctional oligomers for network formation, 35% (v/v) concentrated PGNR solution as photothermal nanoparticle, and 5% (v/v) Darocur 1173 (Sigma-Aldrich) as a photoinitiator.

### *In vitro* Temperature Measurement

Pure DI water was prepared for control experiments, and GNR@MC and DI water mixture was also prepared. 0.5 mL (wet volume) of packed GNR@MCs were added to a cuvette containing 1.5 mL of DI water. Then each sample was irradiated with the 808-nm diode laser with a 2-mm beam diameter for 10 minutes at 20 W/cm^2^. The temperature elevations were measured using a thermo-coupled multimeter (FLUKE 289, Everett, WA, USA).

### Calculation of Heat Conversion Efficiency (*η*)

The heat conversion efficiency of GNR@MC was calculated by modifying previous reports of the solution state^36^. Detailed equations are given as below.

A total energy balance on the system consisting of GNR@MCs in vial irradiated by a NIR laser gives

1 $$\sum _{i}\,{m}_{i}{C}_{p,i}\frac{dT}{dt}={Q}_{P}+{Q}_{dis}-{Q}_{surr}$$

where *m*_*i*_ and *C*_*p*,*i*_ are the mass and heat capacity of the solution, *T* is solution temperature, and *t* is time. *Q*_*P*_ is the photothermal energy inputted by the particle, and this *Q*_*P*_ term can be represented by

2 $${Q}_{P}=I(1-{10}^{-{A}^{808}})\eta $$

where *I* is the incident laser power, *A*^808^ is the absorbance at 808 nm, η is the heat conversion efficiency.

*Q*_*dis*_ is the baseline energy inputted by the sample cell containing only water, and this *Q*_*dis*_ term can be represented by

3 $${Q}_{dis}={m}_{w}{C}_{p,w}$$

where *m*_*w*_ is the mass of water and *c*_*p*,*w*_ is heat capacity of water.

*Q*_*surr*_ is the energy conduction away from the system by air which is nearly proportional to the linear thermal driving force as the proportionality constant

4 $${Q}_{surr}=hS(T-{T}_{surr})$$

where *h* is the heat transfer coefficient, *S* is the surface are of the container, and *T*_*surr*_ is the ambient temperature of the surrounding.

A dimensionless temperature θ is introduced where the *T*_*max*_ denotes the maximum temperature before turning off the NIR light, where the equation (6) is satisfied.

5 $$\theta (t)=\frac{T(t)-{T}_{surr}}{{T}_{max}-{T}_{surr}}$$

6 $${Q}_{P}+{Q}_{dis}=hS({T}_{max}-{T}_{surr})$$

Substituting (5) into (1), and equation (1) can be rearranged by

7 $$\frac{d\theta }{dt}=\frac{hS}{{\sum }_{i}\,{m}_{i}{C}_{p,i}}[\frac{{Q}_{P}+{Q}_{dis}}{hS({T}_{max}-{T}_{surr})}-\theta ]$$

At the cooling stage, that is, the NIR light is shut off, both the *Q*_*P*_ and *Q*_*dis*_ becomes zero, accordingly, the equation (7) changes to the equation (8).

8 $$\frac{d\theta }{\theta }=-\,\frac{hS}{{\sum }_{i}\,{m}_{i}{C}_{p,i}}dt$$

By integrating equation (7), the equation (8) can be derived.

9 $$t=-\,\frac{{\sum }_{i}\,{m}_{i}{C}_{p,i}}{hS}\,\mathrm{ln}\,\theta $$

Therefore, we can get *hS* by applying the linear fitting from the time of the laser shut off state versus ln *θ*.

Thus, the heat conversion efficiency *η* can be calculated by substituting equation (6) for *Q*_*P*_ into equation (2) and rearranging to get

10 $$\eta =\frac{hS({T}_{max}-{T}_{surr})}{I(1-{10}^{-{A}^{808}})}$$

The absorbance of the 0.5 ml GNR@MCs in 2 ml total solution (0.1974), the temperature difference (8.7 K), NIR light intensity (20 W/cm^2^), heat capacity (~8.3628J/K) were applied to the equation (10) to estimate the photothermal transduction efficiency.

### Bio-availability of the GNR@MCs

The cytotoxicity of GNR@MCs for MDA-MB-231 cells was evaluated by the live cell imaging system (DMI6000B, Leica Microsystems, Heidelberg, Germany). MDA-MB-231 were seeded into 96-microwell plate with a density of 2 × 10^4^ cells/well in RPMI1640 (Gibco, Carlsbad, CA, USA) medium supplemented with 10% fetal bovine serum (FBS; Gibco) at 37 °C in a 5% CO_2_ humidified atmosphere. After 24 h incubation, Cells were exposed to varying diluted concentrations of PGNR and GNR@MC for another 20 h. After the washing of suspended GNR@MC by 1X phosphate buffered saline (PBS; Sigma-Aldrich), viable cells were counted in 0.3 × 0.2 mm^2^ area through ImageJ software. The relative cell viability of non-treated control cells was determined and shown as an average ± standard deviation (n = 3).

### *In vitro* Photothermal ablation of cancer cells

MDA-MB-231 cells were seeded in 96-well plates (2 × 10^4^ cells/well) for 24 h and then treated with 1 × 10^5^ GNR@MCs. A diameter of 1 mm region of cells was irradiated with a NIR coherent diode laser (808 nm, 20 W/cm^2^) for 10 min. The photothermal ablation effect on cancer cells of GNR@MC was evaluated by LIVE/DEAD® Viability/Cytotoxicity Kit (Thermo Fisher Scientific, Waltham, MA, USA). For live/dead assay, cells were rinsed with PBS to remove suspended GNR@MC and stained with 1 µM ethidium homodimer-1 (EthD-1, Death cells) and 5 µM calcein AM (Survival cells). The fluorescence signals were visualized by DMI6000B (Leica Microsystems) and the LAS X software (ver. 1, 1, 12420, 0, Leica Microsystems) for processing of captured images.

### Preparation of tumor-bearing mouse model

All experiments were conducted with the approval of the Association for Assessment and Accreditation of Laboratory Animal Care (AAALAC) International, in accordance with the relevant guidelines and regulations. To establish orthotopic tumor model, MDA-MB-231 cells (1 × 10^7^ cells) were inoculated into the proximal thigh of six-week-old BALB/c-nude mice. For minimizing auto-fluorescence and improving image clarity, the mice were fed for a week with alfalfa-free diet.

### Characterization of Cy5.5-conjugated MC and GNR@MC

The absorbance and luminescence spectra of the Cy5.5-conjugated MCs, Cy5.5-conjugated GNR@MCs, and Cy5.5 were measured by using SpectraMax iD5 (Molecular Device). The luminescence spectra were obtained under the excitation wavelength: 650 nm.

### Monitoring the *in vivo* characteristics of Cy5.5-conjugated MC and GNR@MC

After tumor formation, an equal volume of Cy5.5-conjugated MC and GNR@MC (MC-Cy5.5 and GNR@MC-Cy5.5) solution was injected at tumor site for tracking bio-distribution. After implantation of the MC-Cy5.5 and GNR@MC-Cy.5, the mice were scanned using eXplore Optix *in vivo* imaging system (ART Advanced Research Technologies, Montreal) for NIR fluorescence imaging. Optical imaging was performed with a Cy5.5 filter set, excitation wavelength: 670 nm, emission wavelength: 690 nm. The images were acquired for 120 min at 30 min intervals and up to 7 days for long-term. At day 7, the organs of mice were dissected and scanned using eXplore Optix *in vivo* imaging system.

### *In vivo* Photothermal ablation using NIR laser

The Cy5.5-conjugated MC and GNR@MC were injected at the designated sites of tumor-bearing mice with the volume of 60 μl (~20,000 MCs). The laser irradiation was conducted for 30 min at 20 W/cm^2^. Subsequently, the mice were sacrificed, and the laser-irradiated tumor and skin covering tumor were excised and fixed using a 4% paraformaldehyde solution. Histological evaluation was conducted using hematoxylin and eosin (H&E) staining. Tissues were embedded in paraffin after being dehydrated by increasing alcohol concentrations and cleared in xylene. Slices (thickness = 10 μm) were mounted onto glass slides, and for nuclear staining, slides were placed twice in a container filled with hematoxylin for 10 minutes. Tissues were rinsed in water for 10 min to remove hematoxylin, and the cytoplasm was stained with eosin and dehydrated in the same manner as described above. After washing three times for 30 minutes, 2 or 3 drops of the sample were added to the slide and then covered with a cover glass.

## Electronic supplementary material

Supplementary Information

## Data Availability

The datasets generated and/or analyzed during the current study are available from the corresponding author on reasonable request.
