# Supplementary material for: Implantable Photothermal Agents based on Gold Nanorods-Encapsulated Microcube
Source: Sci Rep. 2018 Sep 12;8:13683. doi: 10.1038/s41598-018-31793-9 (PMC6135770; doi:10.1038/s41598-018-31793-9)
Supplement: Supplementary file 1 — Supplementary Information [file 41598_2018_31793_MOESM1_ESM.doc]

**Supplementary Information**

Implantable Photothermal Agents based on Gold Nanorods-Encapsulated Microcube

Hyun June Moon^1,†^, Minhee Ku^2,3,4,†^, Hyun Jee Lee^1^, Nara Yoon^2,3,4^, Jaemoon Yang^2,3,4^,*,

and Ki Wan Bong^1^,*

^1^Department of Chemical and Biological Engineering, Korea University, Seoul, 02841,
Republic of Korea.

^2^Department of Radiology, College of Medicine, Yonsei University, Seoul, 03722,

Republic of Korea

^3^Systems Molecular Radiology, Yonsei University, Seoul, 03722, Republic of Korea

^4^Research Institute of Radiological Science, Yonsei University, Seoul, 03722,

Republic of Korea

(^†^These authors contributed equally to this work.)

**Table of Contents**

1. Validation of homogeneity of gold nanorods (GNRs)-mixed precursor ______________ (3p)
2. GNR encapsulation efficiency in microcubes (MCs) ____________________________ (4p)
3. GNR encapsulation stability in the MCs ______________________________________ (6p)
4. Optimization of flow lithography operating conditions for GNR@MC synthesis______ (8p)
5. Bio-availability of the GNR@MCs __________________________________________ (9p)
6. *In vitro* cancer cell ablation results (cell morphologies) _________________________ (10p)
7. Characterization of optical properties of Cy5.5-conjugated MCs__________________ (11p)
8. *In vivo* tissue ablation test ________________________________________________ (12p)
9. Histological analysis of skin tissues extracted mouse model _____________________ (13p)
10. **Validation of homogeneity of gold nanorods (GNRs)-mixed precursor**


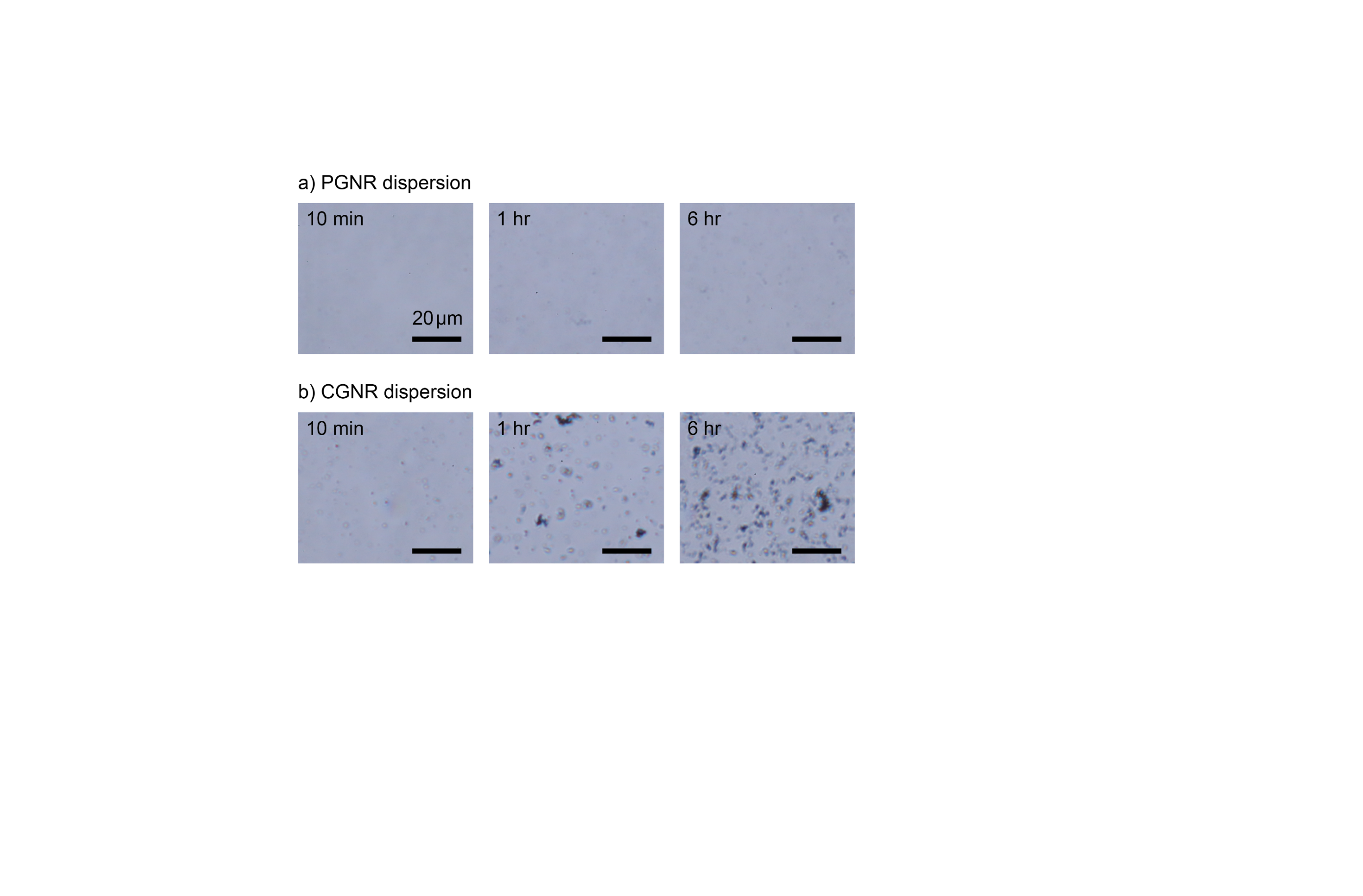


**Figure S1.** Dispersion of GNRs inside the pre-polymer solution. a) GNRs capped with poly(ethylene glycol) (PGNRs) show relatively stable dispersion for 6 h, whereas b) GNRs capped with cetyltrimethylammonium bromide (CGNRs) show remarkable flocculation due to the unstable dispersion.

Optical microscopic (400X) images were taken at specific times by using Inverted Microscope (ZEISS). Both mixtures contain roughly same GNR concentration in unit volume, and the figures below show the difference in flocculation dynamics between poly(ethylene glycol) capped GNRs (PGNRs) and cetyltrimethylammonium bromide capped GNRs (CGNRs). The PGNR mixed precursor shows relatively homogeneous dispersion without significant flocculation for an hour, while the CGNR almost instantly flocculate in the precursor solution.

1. **GNR encapsulation efficiency in microcubes (MCs)**

Encapsulation rate of GNRs during stop flow lithography (SFL) is estimated by comparing the actual loaded mass of GNRs, which is measured by inductively coupled plasma atomic emission spectroscopic (ICP-AES), with the theoretical loading amount. Given the gold mass balance listed below, the fraction of captured GNRs is estimated.

**Gold mass balance calculation**

***1) PGNR suspension: Gold mass per unit volume in the PGNR solution***

- Gold seed solution:

250×10^-6^ L × 10×10^-3^ mol/L (Au) × 196.97 g/mol (Au) = 4.9243×10^-4^ g (Au) inside 8.35 ml solution (250×10^-3^ + 7.5 + 600×10^-3^ ml), yields 5.8974×10^-5^ g/ml Au.

- Growth solution:

50×10^-6^ L × 10×10^-3^ mol/L (Au) × 196.97 g/mol (Au) = 9.8485×10^-5^ g (Au) dispersed in medium of 5 ml solution.

- 5 ml of product (PGNR):

5.8974×10^-5^ g/ml Au × 12×10^-3^ ml (=7.07677×10^-7^ g Au) from 12 μl of gold seed solution and 9.8485×10^-5^ g from the growth solution, the summation yields 9.91927×10^-5^ g Au, (in g/ml unit) **1.98385×10^-5^ g/ml Au.**

***2) Pre-polymer solution: Gold mass per unit volume in the pre-polymer solution***

- (1ml base) precursor solution compositions with 3.5 fold concentrated PGNR and 35 % v/v PGNR solution in 1 ml precursor:

0.35×3.5×1.98385×10^-5^ g/ml Au in 1ml solution, yields **2.4302×10^-5^ g/ml Au.**

***3) Theoretical encapsulation: Gold mass per imaginary volume which is identical volume with that synthesized via stop flow lithography***

- Imaginary volume tantamount to one microcube:

50×50×42.5×10^-18^ m^3^, (in ml unit) 50×50×42.5×10^-12^ ml, yields 1.063×10^-7^ ml per MC

- Theoretical encapsulation of GNRs in the imaginary volume:

2.4302×10^-5^ g/ml Au × 1.063×10^-7^ ml per MC = 2.74×10^-12^ g Au per MC, yields approximately **2.583 pg Au per MC.**

***4) Estimation of GNR loading efficiency based on gold mass balance***

Actual encapsulation mass of gold was estimated based on inductively coupled plasma atomic emission spectroscopic (ICP-AES) analysis. It is estimated that gold encapsulation per each cube is approximately **2.561 pg Au per MC.**

Comparing the actual encapsulation with theoretical amount, the encapsulation efficiency is estimated as ~99.14 %, suggests nearly perfect encapsulation.

1. **GNR encapsulation stability in the MCs**


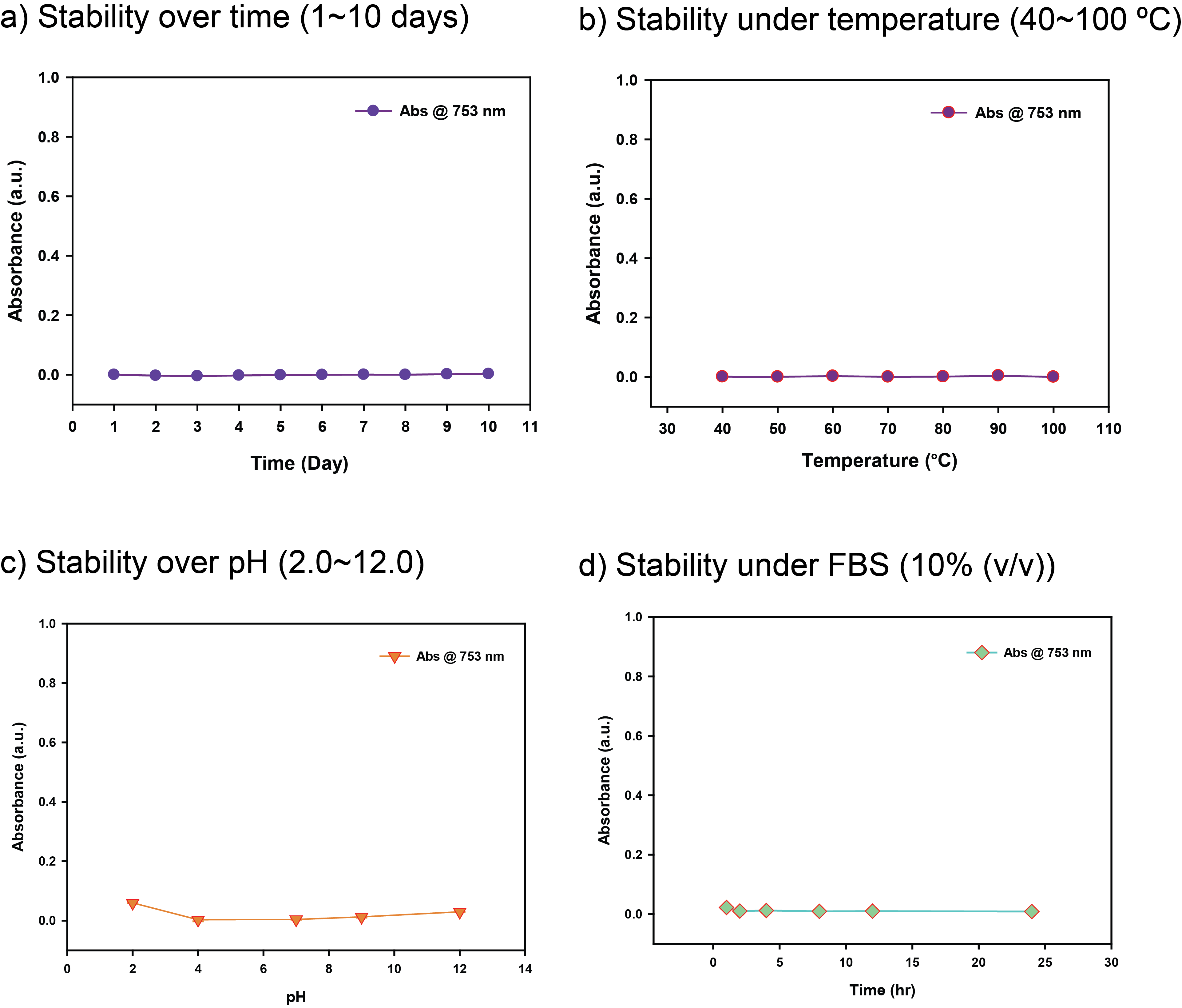


**Figure S2.** Validation of firm encapsulation of GNRs inside the GNR@MCs. a) Encapsulation stability of GNR@MCs over time (temperature: 37 °C), GNRs are shown to be firmly encapsulated inside the GNR@MCs. b) Encapsulation stability of GNR@MCs over temperature (elevated stepwise from 40 to 100 °C. c) Stability under diverse pH (ranging from 2.0 to 12.0), for 24 hours. d) Stability under 10% (v/v) fetal bovine serum (FBS) solution for 1, 2, 4, 8, 12, and 24 hours. The tube was sealed with paraffin film to reduce vaporization of supernatant solution.

To certify that GNRs are firmly encapsulated in the polymeric network inside the particles, we monitored differences in concentration of GNRs in the microcubes-dispersed solution. After slightly centrifuging microcubes-dispersed solution so that we can recover supernatant solution that is free of microcubes. The difference in GNR concentration in the supernatant is monitored for 10 days at physiological temperature (37 °C), when subjected in diverse temperature (40 ~ 100 °C) or pH (2.0 ~12.0), and when submerged in 10% (v/v) fetal bovine serum (FBS). The figures below reveal that GNRs are thoroughly embedded, demonstrated by negligible changes (i.e., increase) in GNR concentration in the supernatant. Two absorbance peaks of GNRs were measured by using UV-vis-NIR spectrometer (TECAN).

1. **Optimization of flow lithography operating conditions for GNR@MC synthesis**

**

**

**Figure S3.** GNR@MC synthesis conditions during flow lithography. a) Photomask design for synthesizing uniform MCs without particles’ mutual sticking nor adhesion to channel walls, b) precursor flow rate estimation based on the migration distance of MCs on the centerline, c) GNR recovery process to increase effective GNR encapsulation by means of recycling, and d) absorbance of recovered GNR compared with that of intact GNR.

1. **Bio-availability of the GNR@MCs**

**
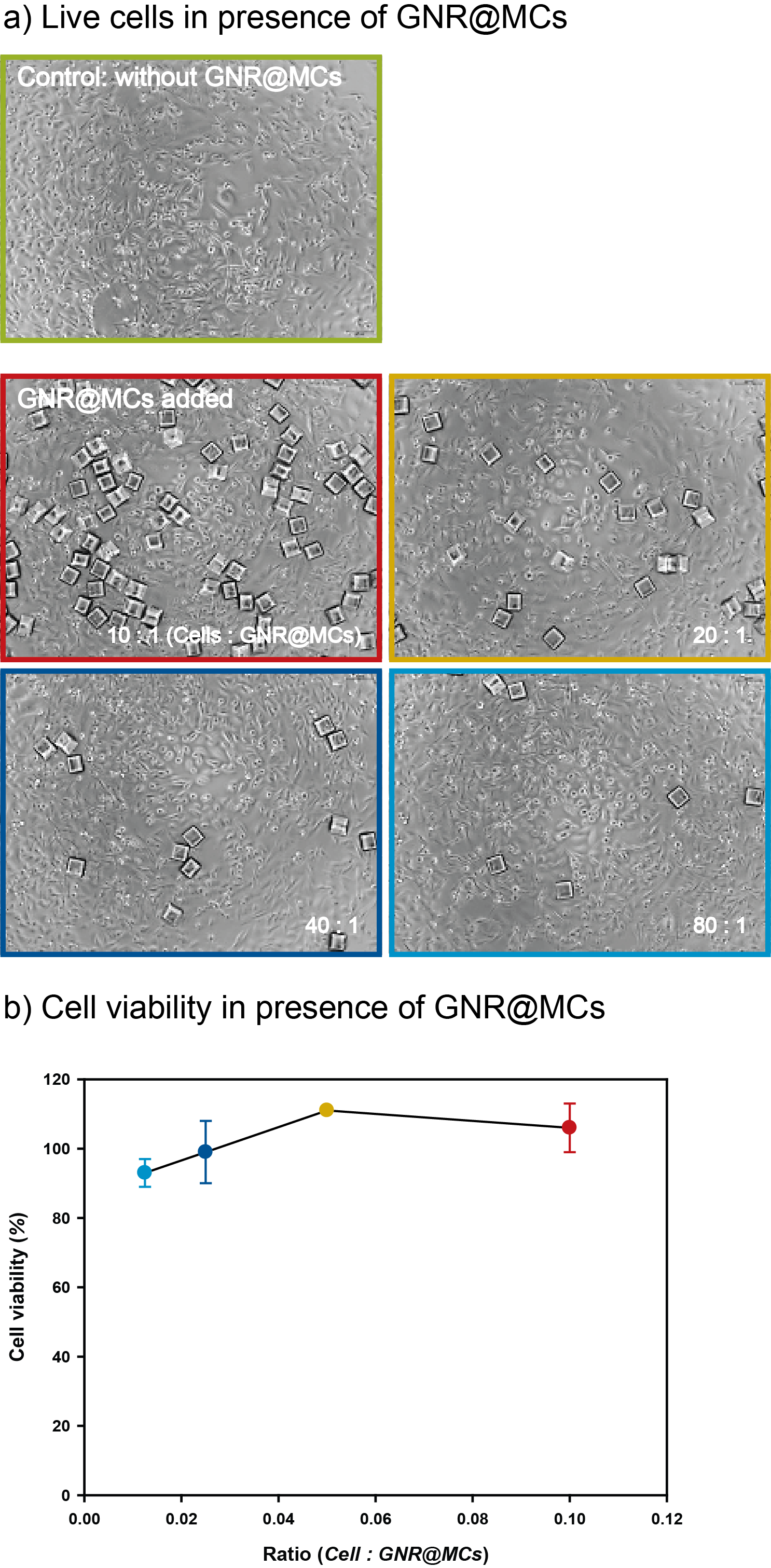
**

**Figure S4.** Bio-availability of the GNR@MCs. a) Live cell (MDA-MB-231) images after co-culture with GNR@MCs of diverse cell-to-MC ratios, b) cell viability after 20 hours. The viable cells were counted in 0.3 x 0.2 mm2 squares through ImageJ software.

1. ***In vitro* cancer cell ablation results (cell morphologies)**


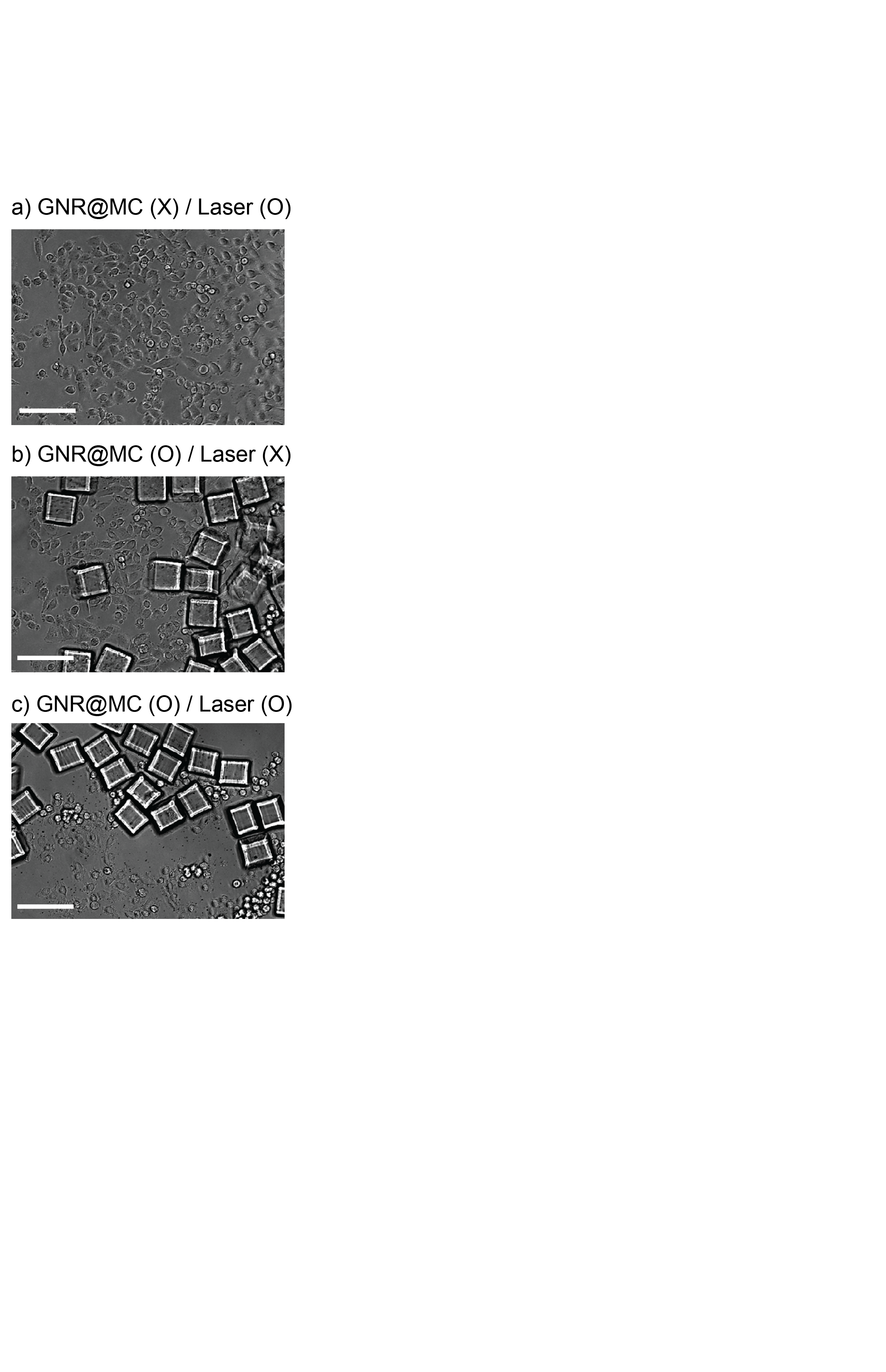


**Figure S5.** *In vitro* cancer cell ablation test. a, b) Cells do not show any significant morphology changes under NIR exposure only or the presence of GNR@MC only, c) cells show a significant morphology change when subjected to both GNR@MCs and NIR exposure. All scale bars are 100 μm.

**7. Characterization of optical properties of Cy5.5-conjugated MCs**


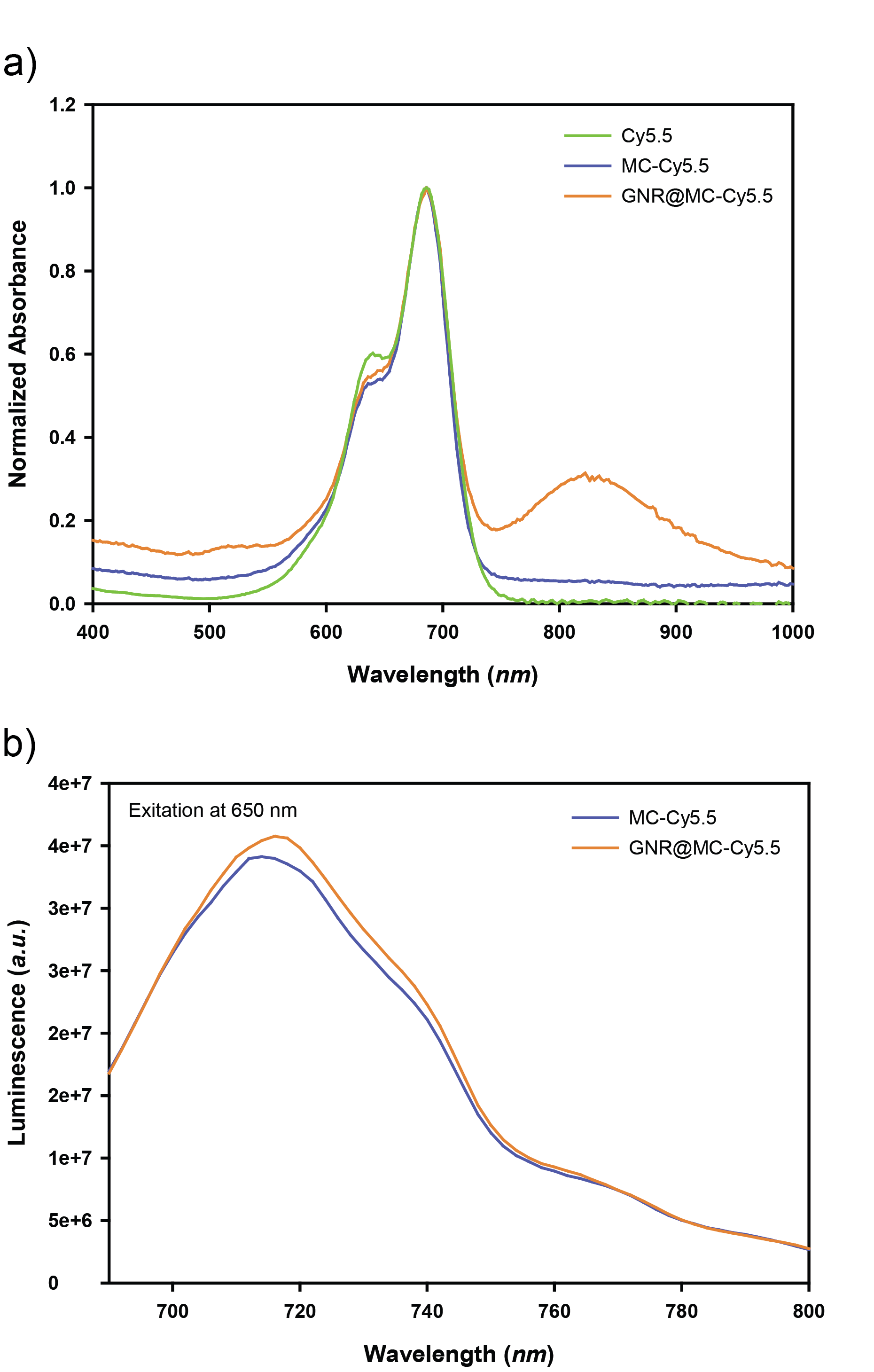


**Figure S6. Optical properties of Cy5.5-conjugated MCs.** a) the normalized absorbance spectra of the Cy5.5 dye, Cy5.5-conjugated MCs (GNR@MCs and plain MCs), b) the luminescence spectra of the Cy5.5-conjugated MCs (Excitation: 650 nm).

**7. *In vivo* tissue ablation test**

**
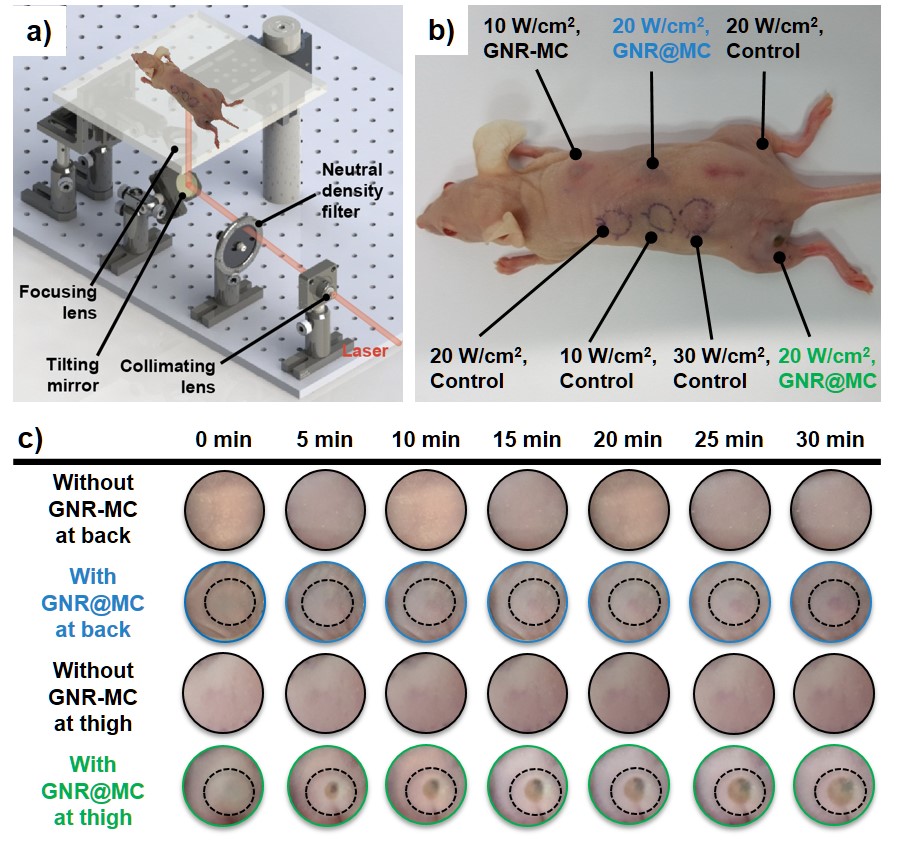
**

**Figure S7. *In vivo* tissue ablation test.** a) the optical system for the photothermal experiment in this study. b) A mouse model for *in vivo* iPT. c) the images taken from a mouse model with or without GNR@MC at the back or thigh after the indicated laser irradiation time. The power intensity of laser was 20 W/cm^2^.

**8. Histological analysis of skin tissues extracted mouse model**

**
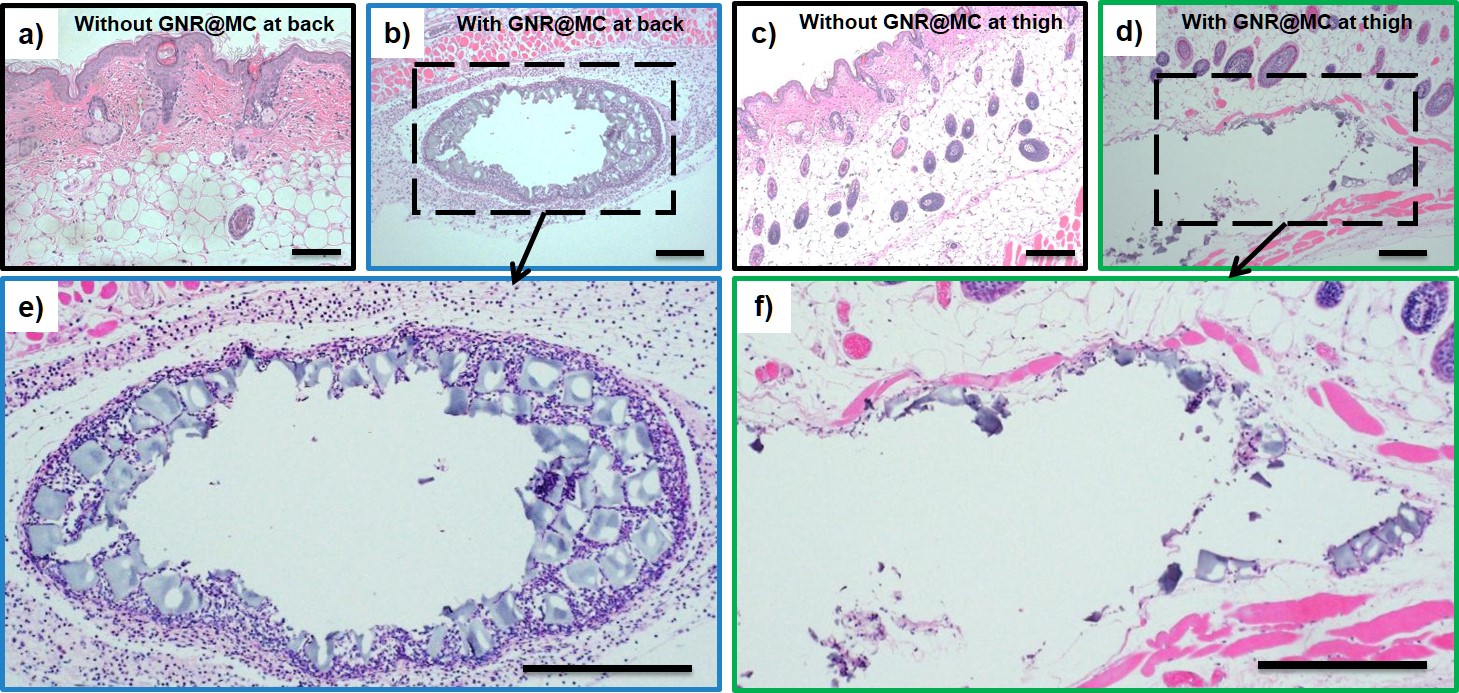
**

**Figure S8. Histological analysis of skin tissues extracted mouse model.** The microscopic images of tissues stained with hematoxylin and eosin (H&E) extracted from the mouse model. a) without GNR@MC at back, b) with GNR@MC at back, c) without GNR@MC at thigh, and d) with GNR@MC at thigh. Magnified microscopic tissue images with GNR@MC e) at the back and f) at the thigh corresponding to b) and d), respectively. All scale bars are 200 µm.
